# Supplementary material for: Are carbon emissions trading and green financial instruments synergistic? -Comprehensive quantitative research based on content analysis
Source: PLoS One. 2024 Mar 7;19(3):e0298601. doi: 10.1371/journal.pone.0298601 (PMC10919601; doi:10.1371/journal.pone.0298601)
Supplement: S1 Data — (ZIP) [file pone.0298601.s001.zip › Data/地级市Z_F工作报告.zip!/╒π╜¡/║■╓▌╩╨/└┤╫╘╣1⁄2╓┌║┼í╛┬φ┐╦╩2╛▌═°í┐_2015.docx]

各位代表：

现在，我代表市人民政府，向大会作工作报告，请予审议，并请市政协委员和其他列席人员提出意见。

　　一、2014年主要工作

过去的一年，宏观经济环境错综复杂，改革发展任务艰巨繁重。我们在省委、省政府和市委的坚强领导下，在市人大、市政协的监督和支持下，紧紧依靠全市人民的共同努力，积极应对挑战，奋力攻坚克难，统筹稳增长、抓改革、调结构、惠民生，经济社会发展稳中有进，现代化生态型滨湖大城市建设迈出新步伐。地区生产总值达到1956亿元，增长8.4%；完成财政总收入295.7亿元，其中地方财政收入167.8亿元，分别增长8.9%和8.5%；预计节能减排各项约束性指标顺利实现。十方面民生实事项目全面完成，城镇居民人均可支配收入达到38959元，农村居民人均可支配收入22404元，分别增长9%和10.6%。我市成为全国地级市中首个生态文明先行示范区，获批国家历史文化名城。

　　（一）突出稳增促调、提质增效，经济运行稳中向好

企业培育工作全面加强。启动“金象金牛”大企业和“高技术、高成长”企业两项培育计划，推动企业主体转型升级， 6家企业入围中国民营企业500强， 12家企业入围制造业500强。改善中小微企业成长环境，完成“个转企” 2428家、“小升规” 230家、“下转上”464家。开展工业、服务业两大帮扶行动，全面走访规模和限额以上企业，协调解决实际问题2500多个。强化金融服务实体经济，金融机构贷款余额新增217.6亿元，银行业不良贷款率由2.02%下降至1.58%。完成直接融资168亿元，新增多层次资本市场挂牌企业14家、上市公司2家。营造贸易便利化环境，推动企业商业模式创新，完成外贸进出口总额99.9亿美元，其中出口88.1亿美元，分别增长4.8%和8.9%。

项目攻坚行动扎实推进。多管齐下抓招商引资，省外浙商回归到位资金184.2亿元，央企合作实到资金58.6亿元，实到外资9.8亿美元。进一步强化“大好高”导向，建立谋划大产业、招引大项目推进机制，新引进首期固定资产投资10亿元以上项目25个。全力扩大有效投入，完成固定资产投资1242.9亿元，增长16.2%； 103个项目列入省“411”实施计划，三一装备工程机械制造基地、凯蒂猫家园等24个项目建成运营，市重点建设项目完成投资601.3亿元。推进产业平台整合提升，南太湖产业集聚区实现实体化运作，湖州开发区等平台新拓展空间28.9平方公里。

产业升级步伐持续加快。深入实施“四换三名”工程，推动工业细分行业转型升级，大力发展信息经济，促进信息化和工业化深度融合，淘汰428家企业的落后产能，完成技改投资352.1亿元，规模以上工业增加值和利税、利润分别增长9.2%和11.5%、 12.2%。印染、造纸、制革、化工行业整治提升工作全省领先，继装备制造成为千亿级产业后，金属新材、绿色家居产值规模双双突破500亿元，战略性新兴产业增加值增长11.4%。协调推进消费升级和商贸业转型，大力发展电子商务，实现社会消费品零售总额871.2亿元、网络零售额135亿元，分别增长13.7%和57.9%。推进国际生态休闲度假城市建设，乡村旅游创新发展，滨湖度假加快提升，南浔古镇保护和利用进一步提速，全市旅游总收入和门票收入分别增长27.9%和20.3%。河海联运在跨区域合作中迅速扩张，内河集装箱吞吐量增长39.9%。文化产业加快发展，服务贸易高速增长，房地产市场趋于平稳。加快农业转型升级，累计建成粮食生产功能区60万亩、现代农业园区45.2万亩，全国现代生态循环农业发展和新型职业农民培育两个试点扎实推进，基层农业公共服务中心实现乡镇全覆盖，我市在农业现代化发展水平综合评价中居全省首位。

　　（二）突出改革统领、创新驱动，发展活力切实增强

简政放权有序推进。实施“四张清单一张网”改革，政府权力清单、责任清单、专项资金管理清单和企业投资项目核准目录清单公布实施，政务服务网开通运行，市级部门行政权力由9377项精减到3800项。实施审批服务“三联动、全流程”改革，提速普遍达到50%以上。在湖州开发区实行“一站式”集中审批服务，基本实现“审批办事不出区”。推行工商登记制度改革，新注册市场主体30402户。实行四类社会组织直接登记制度，新登记各类社会组织190家。实施以转变政府职能为重点的新一轮机构改革，调整完善了市场监管和市区环境保护管理、农村公路管养以及中心城区城管执法体制。

城乡体制和要素机制改革明显加快。推进农村“三权”确权工作，村级集体资产股份合作制改革全面完成，流转土地经营权登记发证试点稳步实施，宅基地确权登记发证率达到69.9%。德清县城乡体制改革试点取得重大突破，织里镇、新市镇小城市培育试点深入推进，练市镇列入全省第二批小城市培育试点。八里店南片新农村综合改革、南浔城南城乡一体化发展两个试验区建设进一步加快。出台土地要素市场化配置十条以及城镇低效用地再开发、土地使用税调整等政策措施，消化批而未供土地14130亩，盘活存量建设用地10370亩，新增工业用地亩均投资强度提高到269万元，我市被列为全国工矿废弃地复垦利用试点。实施重点用能企业能源消费总量核定管理，对9个行业淘汰类、限制类产能执行用电加价措施。

各位代表！人民对美好生活的向往，就是我们的奋斗目标。今年，我们将继续围绕群众急需急盼，全力办好以下十方面民生实事项目：一是大气治理。全面开展城市扬尘治理，创建绿色施工工地30家；对城市主要道路实行机械化清扫，中心城区机械化清扫率达到80%。完成90个煤堆场整治工作。巩固脱硫改造成果，全面完成热电企业脱硝改造。淘汰高污染燃料小锅炉600台。整治餐饮企业油烟排放，中心城区有证照餐饮企业全部安装油烟净化装置。淘汰黄标车9300辆。改造和淘汰老旧船舶120艘。二是城乡治水。完成246个行政村的农村生活污水治理，新增受益农户6.6万户。完成130公里以上城镇污水配套管网建设。加快推进苕溪清水入湖河道整治工程，完成32公里河道拓浚整治。加快太湖水厂及供水配套管网建设，太湖水厂完成工程量60%，完成和孚菱湖、千金石淙善琏片区的供水配套主干管网建设。三是食品安全。创建放心农贸市场15家；开展农贸市场“农产品检测示范单位”创建活动，建成“示范检测室” 20家；加强对餐饮单位食品安全的监管，建成重点餐饮单位“阳光厨房” 100家。实施校园饮食饮水放心工程，学校大宗食品统一配送或定点采购率达到95%，义务教育学校直饮水工程完成率达到70%。四是居住环境。创建市级美丽乡村35个。对700户农村困难群众危旧房实施改造。新竣工保障性住房6600套。完成小西街历史文化街区修缮整治一期工程。五是公共交通。中心城市外环道路建成通车。新增、更新公交车110辆，新增和优化公交线路35条；新建成农村联网公路130公里，完成农村公路桥梁加固与改造50座，新增港湾式停靠站100个。开展住宅小区停车秩序专项整治，中心城区住宅小区新增停车位5000个，周边道路新增时段性停车位500个。六是养老助残。新建成居家养老服务照料中心200家。为500户困难残疾人家庭免费改造无障碍设施；对3400名贫困且生活不能自理的重度残疾人实行托（安）养服务。七是医疗服务。市区新型农村合作医疗和城镇居民基本医疗保险制度并轨，实现城乡居民基本医疗保险一体化。新（迁）建和改扩建社区卫生服务中心12家、服务站30家；组织二级以上公立医院开展100场“进村入企”医疗卫生服务活动。完成红十字应急救护普及培训5万人以上。八是就业扶困。新增城镇就业4.8万人，帮扶困难人员就业3000人。统一城乡居民最低生活保障标准。九是文化体育。建设农村文化礼堂80个。开展全民体育健身项目公益大培训，新设免费培训点120个，培训8万人次。十是便民服务。农村应急广播体系实现行政村全覆盖。建成“智慧社保”手机服务平台。在城乡社区新建成“E邮柜”等电子商务投递终端300个。

　　四、加快建设法治政府

改革发展任务越是艰巨，政府自身建设越要加强。特别是全面深化法治湖州建设，对深入推进依法行政、加快建设法治政府提出了新的更高要求。我们要切实增强宪法和法律意识，自觉把政府工作和自身建设纳入法治轨道，加快建设职能科学、权责法定、执法严明、公开公正、廉洁高效、守法诚信的法治政府，促进治理体系和治理能力现代化，努力为人民提供优质高效服务。（一）推进政府履职科学化。全面完成新一轮政府机构改革，优化机构设置，精简人员编制，清理规范机关事业单位编外用人。加快政府职能转变，开展向社会组织转移职能改革试点，建立权力清单和责任清单动态调整机制，加强事中事后监管制度建设。探索推进县区综合行政执法改革，坚持严格规范公正文明执法，健全行政执法和刑事司法衔接机制，打造法治化的发展环境。完善全口径预算管理，落实财税改革各项任务。按照严控新增、化解存量、提高绩效的要求，全面加强和规范政府性债务管理。突出科学性、前瞻性和可操作性，谋划重大工程、重大项目、重大政策，切实做好“十三五”规划编制工作。（二）推进行政决策规范化。完善政府议事规则，建立行政机关内部重大决策合法性审查机制，健全重大决策公众参与、专家论证、风险评估、合法性审查、集体讨论决定程序。严格执行重大决策出台前向人大常委会报告、与政协民主协商等各项制度规定。对涉及民生的重大决策事项，决策前全面实施社会稳定风险评估。认真执行行政规范性文件审查、备案规定。健全决策后评估和纠错制度，落实重大决策终身责任追究制度及责任倒查机制。充分发挥政府法律顾问和决策咨询制度重要作用，进一步加强政府法制机构建设。

（三）推进权力运行透明化。自觉接受人大监督、民主监督、司法监督、社会监督、舆论监督，加大行政监察力度，推进审计监督全覆盖，努力形成更加科学有效的权力运行制约和监督体系。强化公共资源交易平台建设，进一步拓展交易范围，理顺监管机制。围绕财政预算、公共资源配置、重大建设项目批准和实施、社会公益事业建设等领域，促进决策、执行、管理、服务、结果“五公开”，加快打造“阳光政府”。推进政府门户网、政务服务网和“湖州发布”微博矩阵、微信平台建设，提高政务公开信息化水平。

（四）推进作风建设长效化。巩固和拓展党的群众路线教育实践活动成果，扎实开展“三严三实”专项教育，防止“四风”问题反弹，形成正风肃纪新常态。进一步加大精文简会力度，完善直接联系服务群众制度，健全村（社区）事务准入机制，继续办好“12345政府阳光热线”。严格落实公务接待、因公出国境等各项管理制度，实施公务用车制度改革，做到“三公”经费只减不增。坚持崇尚务实、狠抓落实，进一步加强政务督查，推进政府服务规范化、精准化、长效化，严厉整治行政不作为、慢作为、乱作为现象。全面加强政府系统廉政建设，严格落实党风廉政建设责任制，认真履行“一岗双责”，坚决查办违纪违法案件，保持惩治腐败高压态势。

各位代表！人民赋予重托，拼搏创造未来。让我们在中共湖州市委的领导下，凝聚全市人民的智慧和力量，扎实工作，奋勇前行，为打造“四个湖州”、加快建设现代化生态型滨湖大城市而努力奋斗！
